# Calpain system protein expression and activity in ovarian cancer

**DOI:** 10.1007/s00432-018-2794-2

**Published:** 2018-11-17

**Authors:** Siwei Zhang, Suha Deen, Sarah J. Storr, Panagiota S. Chondrou, Holly Nicholls, Anqi Yao, Ployphailin Rungsakaolert, Stewart G. Martin

**Affiliations:** 1 Academic Clinical Oncology, Nottingham Breast Cancer Research Centre, School of Medicine, University of Nottingham, Nottingham University Hospitals NHS Trust, City Hospital Campus, Nottingham, NG5 1PB UK; 2 Department of Histopathology, Queen’s Medical Centre, Nottingham University Hospitals NHS Trust, Nottingham, NG7 2UH UK; 3 Present Address: Lungs for Living Research Centre, UCL Respiratory, University College London, 5 University Street, London, WC1E 6JF UK

**Keywords:** Calpain, Calpastatin, Ovarian cancer, Chemotherapy, Epithelial–mesenchymal transition

## Abstract

**Purpose:**

Expression of members of the calpain system are associated with clinical outcome of patients with, amongst others, breast and ovarian cancers, with calpain-2 expression in ovarian cancer being implicated in chemo-resistance and survival. This study aimed, using a large patient cohort and in vitro models, to verify its importance and further investigate the role in ovarian cancer chemoresponse.

**Methods:**

Calpain-1, calpain-2, calpain-4 and calpastatin expression were evaluated in primary ovarian carcinomas (*n* = 575) by immunohistochemistry. Protein expression was assessed, via western blotting, in five ovarian cancer cell lines with various sensitivities towards cisplatin/carboplatin. In vitro calpain activity was inhibited by calpeptin treatment to assess changes in platinum sensitivity by proliferation assay, with expression of genes associated with epithelial–mesenchymal transition being examined by RT^2^ Profiler™ PCR Array.

**Results:**

The current study confirmed previous data that high calpain-2 expression is associated with poor overall survival (*P* = 0.026) and that calpain-1 was not associated with overall survival or progression-free survival. Low expression of calpastatin (*P* = 0.010) and calpain-4 (*P* = 0.003) were also associated with adverse survival. Such prognostic associations do not seem to be linked with altered tumour sensitivity towards platinum-based chemotherapy. Interestingly, low calpain-1 expression was more frequent in patients with confined tumours (stage 1) (*χ*^2^ = 11.310, *df* = 1, *P* = 0.001). Calpain and calpastatin expression varied among ovarian cancer cell lines yet their expression levels were similar between chemo-sensitive cells and resistant counterparts. Moreover, calpeptin treatment did not alter cellular response to platinum-based chemotherapy or epithelial–mesenchymal transition-related gene expression.

**Conclusions:**

The conventional calpains and calpastatin have been confirmed to play an important role in ovarian cancer; however, the precise mechanisms whereby they exert effects remain to be elucidated.

**Electronic supplementary material:**

The online version of this article (10.1007/s00432-018-2794-2) contains supplementary material, which is available to authorized users.

## Introduction

Calpains, calcium-dependent papain-like enzymes, are cytoplasmic neutral cysteine proteases that regulate various cellular processes, such as signal transduction, cytoskeletal organisation, cell survival and cell death; via cleavage of numerous substrates (Storr et al. 2011a). Although there are 16 calpains, the ubiquitously expressed conventional calpain subunits are the most widely studied. Conventional calpains include micro-calpain (µ-calpain) and milli-calpain (m-calpain), which are functional heterodimeric calpains. The heterodimer consists of one calpain large subunit, i.e. calpain-1 or calpain-2 and a common small subunit, i.e. calpain-4 (Storr et al. 2011a). The activation of the conventional calpains is tightly controlled by multiple mechanisms including calcium influx, the relative abundance of calpain subunits and of their endogenous inhibitor, calpastatin (Zhang et al. 2017).

The relationship between the expression of the calpain system in cancer and patient outcome has been examined in a number of studies. Whether expression and outcomes are significantly associated or whether the associations were positive or negative seems to depend on context, such as tumour type, subtype and heterogeneity (Storr et al. 2011b, c, 2012a, b; Al-Bahlani et al. 2017). By comparing matched chemo-sensitive and chemo-resistant cell lines, calpains have been suggested to be an important protein family involved in the cellular response to chemotherapy. Calpain-2 has been found to be overexpressed both at the mRNA and protein levels in chemo-resistant colon cancer HT-29R cells in comparison with the parental line (Fenouille et al. 2012). Calpain activity was significantly higher in cisplatin-resistant MeWo-cis1 cells than in the parental MeWo cells (Młynarczuk-Biały et al. 2006), and calpain-4 expression was shown to be significantly lower in cisplatin-resistant gastric cancer SGC-7901/DDP and BGC-823/DDP cells than in parental cells (Zhang et al. 2016).

As indicated above, aberrant expression, or activity, of the calpain system occurs in various cancers. Yet very little information is available regarding the association of calpains and calpastatin expression with clinicopathological factors and prognosis in ovarian cancer. Salehin et al., (2011) indicated that calpastatin expression levels were lower in ovarian tumours compared to normal tissues, whilst high calpain-1 expression was linked with lower tumour grade; with low calpain-2 expression being linked with increased lymph node metastasis. Yang et al. (2017) found a negative association between calpain-4 expression and overall survival with high calpain-4 expression significantly associated with the presence of lymph node metastasis (*P* = 0.009) and high FIGO stage (*P* = 0.001, n = 91). In a previously published study by our group (*n* = 154 patients), high calpain-2 expression was significantly associated with platinum resistance (*χ*^2^ = 4.658, *df* = 1, *P* = 0.031), poor progression-free survival (*P* = 0.049) and poor overall survival (*P* = 0.006) (Storr et al. 2012a). High expression was significantly associated, in multivariate analysis, accounting for grade, stage, optimal debulking and platinum sensitivity, with worse OS (hazard ratio = 2.174; 95% confidence interval = 1.144–4.130; *P* = 0.018) (Storr et al. 2012a). The current study, using a larger patient cohort and including calpain-4 expression, aimed to verify such results and to use in vitro experimental models to examine potential regulatory mechanisms.

## Materials and methods

### Clinical samples

The ovarian tissue microarray was composed of tumour cores from 575 ovarian cancer cases; with 448 chemo-naïve samples and 22 cases sampled post-chemotherapy (no chemotherapy details were available on the remainder). Staging was according to the International Federation of Obstetrics and Gynecology (FIGO) staging system. Grading was performed using the Shimizu–Silverberg grading system. The clinicopathological variables of the cohort are listed in Table 1. Patients were diagnosed with ovarian cancer and received treatment at Nottingham University Hospitals between 1991 and 2011. The median follow-up period was 8 years: ranging between 3 years and 20 years. The median overall survival time was 44 months: ranging from 0 to 223 months. The majority of the patients (*n* = 357) received platinum-based chemotherapy among which 168 patients were treated with chemotherapy containing taxanes (Table S1). Progression-free survival was defined as the length of time between start of treatment and clinical identification of recurrence. Data on the resistance to chemotherapy were recorded, classified by the Gynaecologic Oncology Group (GOG) as either refractory (not responding to chemotherapy), resistant (an initial response to chemotherapy with recurrence within 6 months) or sensitive (when there was either no recurrence or recurred after 6 months). Ethical approval was obtained from Derbyshire Ethics Committee (07/H0401/156). This study is reported in accordance with REMARK (reporting recommendations for tumour marker prognostic studies) criteria (McShane et al. 2005).

**Table 1:** Clinicopathologic variables of patient cohort

Variables	Number of Patients	Percentage (%)
Age		
≤ 62	295	52.4
> 62	268	47.6
Histological subtypes		
HGSC	337	59.7
Mucinous carcinoma	60	10.6
Endometrioid carcinoma	68	12.1
CCC	53	9.4
LGSC	30	5.3
SBOT	15	2.7
Grade		
1	48	8.5
2	90	16.0
3	425	75.5
Stage		
I	203	36.7
II	64	11.6
III	245	44.3
IV	41	7.4
Residual disease		
No residual tumour	311	62.2
Residual tumour (< 2 cm)	58	11.6
Residual tumour (> 2 cm)	131	26.2
Adjuvant therapy		
Pt-based chemotherapy	357	63.3
Non-platinum-based chemotherapy	6	1.1
No chemotherapy	80	14.2
Information not available	121	21.5
Response to chemotherapy		
Refractory and resistance	66	17.7
Sensitivity	307	82.3
Progression status		
No recurrence	137	32.9
Recurred	280	67.1
Survival status		
Living	234	42.0
Deceased	323	58.0

### Tissue microarray, immunohistochemistry and interpretation

Protein expression was investigated using sections taken from tissue microarray blocks, which were constructed as follows. Tumour samples had been immediately fixed in 10% formalin and embedded into paraffin blocks. Donor blocks were sectioned and stained with haematoxylin and eosin with a specialist gynaecological pathologist reviewing and marking the area of interest to core. A Beecher instrument manual tissue microarrayer was used to produce the TMA paraffin blocks by extracting cores of 0.6 mm from the donor blocks and placing them in pre-punched holes in the TMA blocks, each with approximately 160 cores. For the majority of the cases, a single tissue core was used for each patient. There were 48 cases that had two cores per case and 3 that had three cores per case. A small number of cores from other tissues were also placed in each TMA block to act as potential subsequent positive controls and to aid orientation. Fresh sections (4 µm) were cut from each block and placed on coated glass slides for the immunohistochemical assessment of protein expression.

Immunohistochemistry was performed as described previously (Storr et al. 2012a), following re-optimisations. Briefly, slides were heated at 60 °C for 10 min then dewaxed in xylene and rehydrated in industrial methylated spirits. Sections were pre-treated by heat-induced epitope retrieval in 0.01 mol/L sodium citrate buffer, pH 6, for 10 min (750W) + 10 min (450W) in a microwave oven. Staining was achieved using a Novolink Polymer Detection System (Leica, Denmark) following the manufacturers’ instructions. Briefly, endogenous peroxidase activity was neutralised with peroxidase block reagent for 5 min at room temperature; followed by application of protein block reagent for 5 min at room temperature, to minimise non-specific interactions of the subsequent detection reagents after washing with Tris buffered saline. Primary antibody was diluted in bond primary antibody diluent (Leica, Denmark) and applied to the tissue overnight at 4 °C. The primary antibodies were diluted in bond primary antibody diluent (Leica, Denmark), calpain-1 (1:1000, Santa Cruz Biotechnology, INC.), calpain-2 (1:2500, Chemicon^®^ International Millipore), calpain-4 (1:100,000, Chemicon^®^ International Millipore) and calpastatin antibody (1:50,000, Chemicon^®^ International Millipore), and applied to the tissue overnight at 4 °C. The specificity of these antibodies was initially confirmed, by western blotting, before use. Post-primary reagent, a polymer penetration enhancer, was applied on the slides for 30 min; followed by NovoLink Polymer [anti-mouse/rabbit IgG-Poly- horseradish peroxidase (HRP)] for another 30 min, with each step followed by a Tris buffered saline wash. Immunohistochemical reactions were visualised with 3, 3′-diaminobenzidine (DAB) for 5 min and counterstained with haematoxylin for 6 min. Slides were then dehydrated with industrial methylated spirits and xylene and mounted with DPX (SIGMA). Negative controls had primary antibody omitted.

Slides were scanned at high resolution using a Nanozoomer Digital Pathology Scanner (Hamamatsu Photonics) with 200× magnification. Staining intensity was semi-quantitatively assessed using an immunohistochemical H-score with ranking from none (0), weak (1), medium (2) to strong (3) with the percentage area of each staining intensity being multiplied by the intensity rank (H-score range: 0–300). Greater than 25% of the slides were examined by a second independent assessor blinded to scores and clinicopathologic criteria. Single measure intraclass correlation coefficient (ICC) analysis was used to determine the level of agreement between independent scorers. The single measure ICCs between scores were 0.8, 0.814, 0.733 and 0.796 for calpain-1, calpain-2, calpain-4 and calpastatin protein expression, respectively. A non-biased cut-point of the immunohistochemical scores, to dichotomise data, was determined using X-tile software using patient OS (Camp et al. 2004; Storr et al. 2012a).

### Cell culture

Ovarian cancer cell lines PEO1 and PEO4 cells were obtained from the American Type Culture Collection. A2780, A2780-cis and SKOV-3 cells were obtained from the European Collection of Authenticated Cell Cultures. Cells were cultured in Roswell Park Memorial Institute (RPMI)-1640 medium (SIGMA) (for A2780 and A2780-cis), RPMI medium (SIGMA,) containing Sodium Pyruvate (SIGMA) 2 mM (for PEO1 and PEO4) or McCoy’s 5A Modified medium (SIGMA) (for SKOV-3) supplemented with 10% heat-inactivated iron-supplemented donor bovine serum (Gibco, Life Technologies) and penicillin/streptomycin (SIGMA) (with 10,000 units penicillin and 10 mg streptomycin/mL) in 37 °C and 5% CO_2_ atmosphere. Cisplatin (1 µM) was added to A2780-cis culture media every two to three passages. Cell line authentication was conducted using the Promega Powerplex^®^ 16-short-tandem-repeat system. All cell lines were regularly screened for mycoplasma infection using a MycoProbe^®^ Mycoplasma Detection Kit (R&D Systems). Cells in logarithmic growth phase were used for experiments.

### Drug preparation

Stock solution of cisplatin (p4394, SIGMA) was made at 1 mg/ml in sodium chloride solution (0.9%) (S8776, SIGMA). Carboplatin (c2538 SIGMA) was initially diluted in sterile distilled water (dH_2_O) to reach 20 mM. Both stock solutions were protected from light, stored at room temperature and used within 2 months. Calpeptin (03-34-0051, Merck Millipore) was initially diluted to 100 mM in dimethyl sulfoxide (DMSO, D2650, SIGMA), and then the aliquoted stock solution was stored at − 20 °C.

### Western blotting

Subconfluent cells were trypsinised, washed, collected and lysed at 5 × 10^6^ cells per ml in RIPA buffer (R0278, SIGMA) supplemented with 1 × Halt Protease Inhibitor Cocktail containing protease inhibitors, phosphatase inhibitors and EDTA (ThermoFisher Scientific) at 4 °C for 10 min and then lysates were frozen at − 20 °C or − 70 °C for long-term storage.

Cell lysate was loaded into SDS-PAGE gel (Novex® ready-made NuPAGE^®^ 4–12% Bis-Tis Protein Gels), after which proteins were separated by gel electrophoresis and transferred onto a 0.2-µm nitrocellulose membrane (Millipore). Membranes were then blocked with 3% non-fat milk PBS containing 0.1% Tween 20 prior to incubation with primary antibody overnight at 4 °C. Antibodies were used at the following dilutions: anti-calpastatin 1:1000, anti-calpain-1 1:1000, anti-calpain-2 1:2500 and anti-calpain-4 1:1000. Membranes were washed and incubated with mouse/rabbit anti-β-actin antibody [1 in 1000 dilution, ab8226 or ab8227 (Abcam)], for 1 h at room temperature. Secondary antibodies [i.e. 680 Donkey anti-Mouse IgG (H + L) (926-32222, IRDye^®^, LI-COR) and 800CW Donkey anti-Rabbit IgG (H + L) (926-32213, IRDye^®^, LI-COR)] were incubated on the membrane for 1 h. Membranes were visualised using an Odyssey FC Imager (LI-COR Biosciences). The fluorescence intensity was quantified using Image Studio Version 4.0. (LI-COR Biosciences) and normalised against β-actin signals.

### Evaluation of cell proliferation and chemosensitivity

SKOV-3, PEO1 and PEO4 cells were used to assess effects of calpain inhibition on cell proliferation and chemosensitivity. To ensure cells were in log phase during assessment, 2 ml of cell suspension (8 × 10^4^/ml SKOV3 cells, 1 × 10^5^/ml PEO1 cells and 1 × 10^5^/ml PEO4 cells) were seeded into each well of six-well plates. Cells were plated for variable times before treatment, according to particular doubling times. SKOV3 and PEO1 cells were cultured for 1 day whilst PEO4 cells for 3 days before treatment. Doubling time for SKOV3’s was 32.3 ± 4.0 h, for PEO1 57.4 ± 5.7 h and for PEO4 102.7 ± 8.5 h (data not shown). Cells were then pre-treated for 1 h (SKOV3) or 90 min (PEO-1 and -4) with 50 µM calpeptin followed by an additional 48-h treatment with cisplatin (SKOV3: 5 µM, PEO1: 3 µM, PEO4: 19 µM) or carboplatin (SKOV3: 11 µM, PEO1: 14 µM, PEO4: 26 µM) (IC50 concentrations) in the presence of 50 µM calpeptin. Cells in each well were trypsinised and counted at various times thereafter.

### RNA extraction and real-time EMT PCR array

PEO-1/-4 cells were treated with DMSO or 50 µM calpeptin for 4 h or 24 h based on calpain activity results from fluorescent plate reader assays using a fluorescent substrate CMAC, t-BOC-Leu-Met, which indicated that 50 µM calpeptin induced maximal inhibition over a 90 min–48 h treatment time (approximately 30% for PEO1 and 40% for PEO4) (data not shown). Total RNA was extracted from treated cells with RNA protect Cell Reagent (Qiagen) and purified with the RNeasy Plus Mini Kit (Qiagen) according to the manufacturer’s instructions. RNA samples from three independent experiments were pooled together. After quantification using a Nanodrop spectrophotometer (calculation of 260/280 nm ratio), 0.5 µg total RNA was reversed transcribed into cDNA using RT^2^ First Strand Kit (Qiagen) at 42 °C for 15 min following by 95 °C for 5 min to stop the reaction. Real-time PCR was performed using the Human Epithelial to Mesenchymal Transition (EMT) RT^2^ Profiler™ PCR Array (PAHS-090Z, Qiagen) in combination with RT^2^ SYBR Green Mastermixes (Qiagen). The PCR array profiles the expression of 84 key genes involved in EMT process. The PCR cycling condition was set as follows: 95 °C for 10 min, 40 cycles of 95 °C for 15 s and 60 °C for 1 min and run on ABI-7500 (Applied Biosystems). Data were analysed using the ΔΔCt method.

### Statistical analysis

The relationships between categorised protein expression and clinicopathologic factors were examined using Pearson’s chi-square test of association (*χ*^2^) or Fisher’s exact test if a cell count was less than 5 in a 2 × 2 table. To assess the relationship between protein expression and survival outcomes, survival curves were generated using the Kaplan–Meier method and statistical significance determined by the Log-rank test. Multivariate survival analysis was performed using a proportional hazards model by Cox regression analysis to estimate hazard ratios and 95% confidence intervals for overall survival. Spearman’s rank correlation coefficient (Spearman’s rho) test was performed to assess the correlation between the expression levels of different proteins. The correlation strength was interpreted as follows: Spearman rho (rs) less than 0.16 is too weak to be meaningful, ranging from 0.16 to 0.19 as very weak correlation, 0.20 to 0.39 as a weak correlation, 0.40 to 0.59 as a moderate correlation, and 0.60 to 0.79 as strong correlation, and 0.80 or greater as very strong correlation (Divaris et al. 2012). Results from in vitro experiments were represented as average ± standard deviation from a minimum of three independent experiments. For comparing two variables, the Student’s *t* test was used and one-way ANOVA was used to compare three or more groups. Statistical analyses were carried out using SPSS 22.0 software. *P* values < 0.05 were considered statistically significant. Data analysis of the PCR array was carried out using the online GeneGlobe Data Analysis Centre. Fold-change of target genes against the reference gene was calculated from 2^−△△Ct^ values. The CT cut-off was set at 35. Gene expression differences greater than twofold were considered as differentially transcribed.

## Results

### Expression pattern of the calpain system in ovarian cancer

**Fig. 1:**
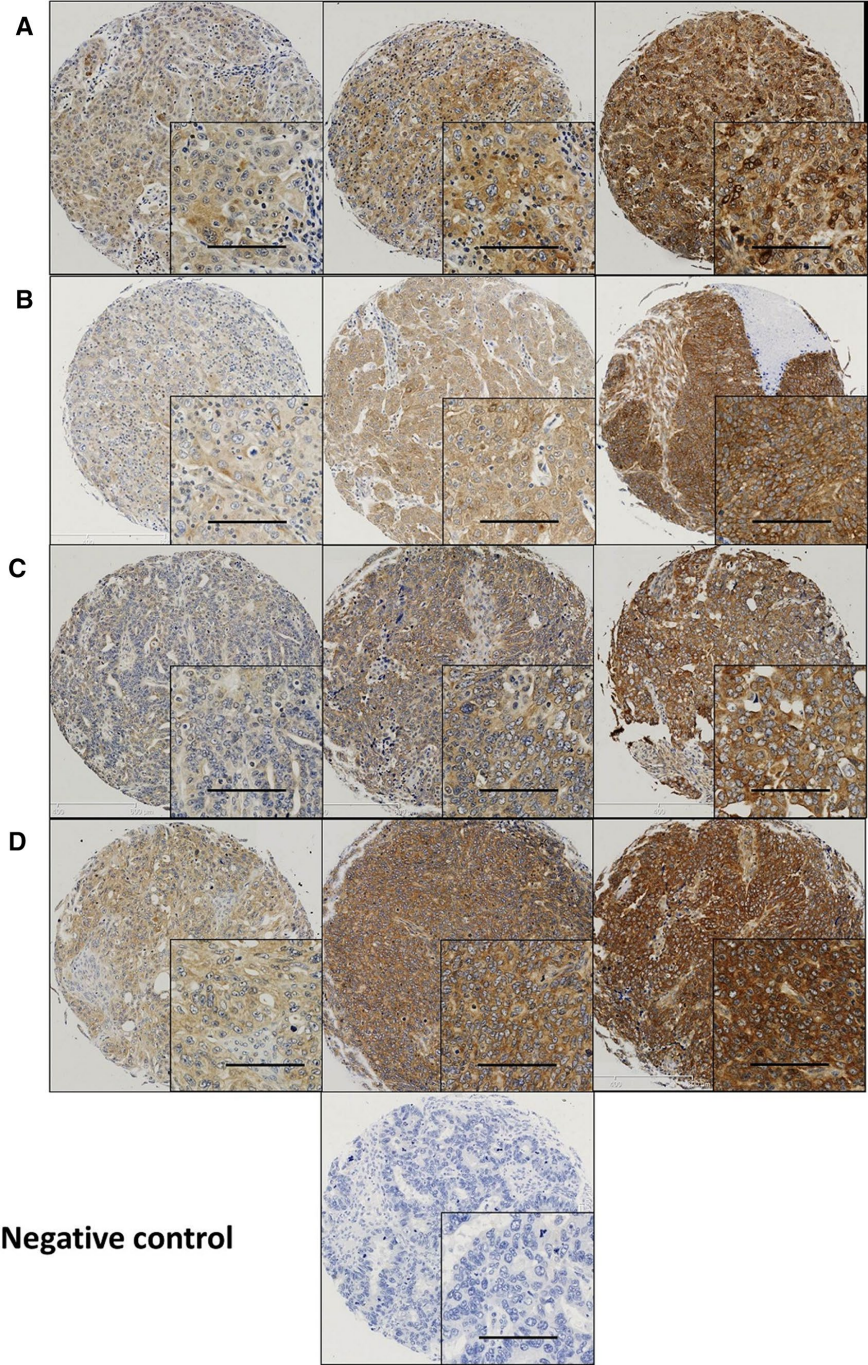
Representative photomicrographs of calpains and calpastatin expression in ovarian carcinoma cells. Expression levels, including low (left), medium (middle) and high (right) staining, of **a** calpastatin, **b** calpain-1, **c** calpain-2 and **d** calpain-4 in the cytoplasm at × 10 magnification with × 20 magnification inset panel. Scale bar represents 100 µm

Calpain-1, -2, -4 and calpastatin expression was confined to the membrane and cytoplasm (Fig. 1). Calpains and calpastatin showed predominant granular/diffuse staining in the cytoplasm of the ovarian cancer cells with heterogeneity between adjacent tumour cells, varying from weak to intense staining. Some stromal cell staining was observed; however, this was not scored as part of this study.

There were 98 cases common to both the current and the previously reported cohort (Storr et al. 2012a). Although tissues were from the same cases, different positions of the respective primary tumour were sampled for each of the two cohorts. Average protein expression from the previous study was compared with the protein expression in matched cases from the current cohort using Spearman’s correlation test. Calpastatin and calpain-1 expression levels were significantly correlated with each other in these two studies (rs = 0.602, *P* < 0.001, *n* = 67 and rs = 0.512, *P* < 0.001, *n* = 69 respectively). No correlation between calpain-2 expression in these two studies (rs = 0.179, *P* = 0.141, *n* = 69) was observed suggesting potential heterogeneity of expression of the calpain system in tumours.

### Calpain system expression and clinicopathological factors

High calpastatin expression was observed in 256 (55%) out of 469 cases (cut-point: 80). Pearson’s chi-squared test was performed to evaluate the relationships between the expression of calpain system and clinicopathologic characteristics with results shown in Table 2. High calpastatin expression was associated with younger patients (*χ*^2^ = 4.955, *df* = 1, *P* = 0.026) and high-grade serous carcinoma (HGSC) (*χ*^2^ = 17.403, *df* = 5, *P* = 0.004) whilst low calpastatin expression was linked with clear-cell carcinoma (CCC). This association between calpastatin and ovarian subtype was also significant in chemo-naïve samples (*χ*^2^ = 15.958, *df* = 5, *P* = 0.007).

**Table 2:** Association between protein expression and clinicopathological variables

Variables	Calpastatin			Calpain-1			Calpain-2			Calpain-4		
	Low	High	*P* value	Low	High	*P* value	Low	High	*P* value	Low	High	*P* value
Age												
≤ 62	97 (20.7%)	143 (30.5%)	0.026	50 (10.7%)	190 (40.5%)	0.705	45 (9.6%)	195 (41.7%)	0.880	67 (14.3%)	173 (36.9%)	0.215
> 62	116 (24.7%)	113 (24.1%)		51 (10.9%)	178 (38.0%)		44 (9.4%)	184 (39.3%)		76 (16.2%)	153 (32.6%)	
Histological subtypes												
HGSC	122 (26.0%)	165 (35.2%)	0.004	38 (8.1%)	249 (53.1%)	< 0.001^a^	54 (11.5%)	233 (49.8%)	0.334^a^	83 (17.7%)	204 (43.5%)	0.003a
Mucinous	24 (5.1%)	21 (4.5%)		14 (3.0%)	31 (6.6%)		11 (2.4%)	34 (7.3%)		14 (3.0%)	31 (6.6%)	
Endometrioid	25 (5.3%)	30 (6.4%)		20 (4.3%)	35 (7.5%)		6 (1.3%)	48 (10.3%)		14 (3.0%)	41 (8.7%)	
CCC	31 (6.6%)	13 (2.8%)		25 (5.3%)	19 (4.1%)		12 (2.6%)	32 (6.8%)		25 (5.3%)	19 (4.1%)	
LGSC	7 (1.5%)	17 (3.6%)		3 (0.6%)	21 (4.5%)		3 (0.6%)	21 (4.5%)		5 (1.1%)	19 (4.1%)	
SBOT	4 (0.9%)	10 (2.1%)		1 (0.2%)	13 (2.8%)		3 (0.6%)	11 (2.4%)		2 (0.4%)	12 (2.6%)	
Grade												
1	16 (3.4%)	21 (4.5%)	0.796	8 (1.7%)	29 (6.2%)	0.415	8 (1.7%)	29 (6.2%)	0.915	9 (1.9%)	28 (6.0%)	0.196
2	31 (6.6%)	42 (9.0%)		20 (4.3%)	53 (11.3%)		14 (3.0%)	59 (12.6%)		17 (3.6%)	56 (12.0%)	
3	166 (35.5%)	192 (41.0%)		73 (15.6%)	285 (60.9%)		67(14.3%)	290 (62.1%)		117 (25.0%)	241 (51.5%)	
Response to platinum-based chemotherapy												
Pt sensitive	96 (%)	121 (%)	0.278	46 (%)	171 (%)	0.854	45 (%)	171 (%)	0.630	62 (%)	155 (%)	0.644
Pt resistance	14 (%)	26 (%)		9 (%)	31 (%)		7 (%)	33 (%)		10 (%)	30 (%)	
Stage												
I	83 (18.0%)	86 (18.7%)	0.217	51 (11.1%)	118 (25.6%)	0.002	35 (7.6%)	133 (28.9%)	0.572	52 (11.3%)	117 (25.4%)	0.969
II	24 (5.2%)	30 (6.5%)		13 (2.8%)	41 (8.9%)		7 (1.5%)	47 (10.2%)		17 (3.7%)	37 (8.0%)	
III	82 (17.8%)	123 (26.7%)		28 (6.1%)	177 (38.4%)		40 (8.7%)	165 (35.9%)		59 (12.8%)	146 (31.7%)	
IV	18 (3.9%)	15 (3.3%)		8 (1.7%)	25 (5.4%)		5 (1.1%)	28 (6.1%)		10 (2.2%)	23 (5.0%)	
Stage												
Confined tumour (I)	83 (18.0%)	86 (18.7%)	0.167	51 (11.1%)	118 (25.6%)	0.001	35 (7.6%)	133 (28.9%)	0.425	52 (11.3%)	117 (25.4%)	0.766
Tumour spread (II–IV)	124 (26.8%)	168 (36.4%)		49 (10.6%)	243 (52.7%)		52 (11.3%)	240 (52.2%)		86 (18.7%)	206 (44.7%)	
Distant metastasis												
Absence (stage I–III)	189 (41.0%)	239 (51.8%)	0.248	92 (20.0%)	336 (72.9%)	0.712	82 (17.8%)	345 (75.0%)	0.567	128 (27.8%)	300 (65.1%)	0.962
Presence (stage IV)	18 (3.9%)	15 (3.3%)		8 (1.7%)	25 (5.4%)		5 (1.1%)	28 (6.1%)		10 (2.2%)	23 (5.0%)	
Residual disease												
No residual tumour	114 (27.5%)	143 (34.5%)	0.958	73 (17.6%)	184 (44.3%)	< 0.001	55 (13.3%)	201 (48.6%)	0.677	76 (18.3%)	181 (43.6%)	0.982
Residual tumour < 2 cm	23 (5.5%)	27 (6.5%)		5 (1.2%)	45 (10.8%)		11 (2.7%)	39 (9.4%)		15 (3.6%)	35 (8.4%)	
> 2 cm	47 (11.3%)	61 (14.7%)		14 (3.4%)	94 (22.7%)		19 (4.6%)	89 (21.5%)		33 (8.0%)	75 (18.1%)	

*Pt* Platinum

^a^Have expected count less than 5. Significant *P* values are indicated by bold type

High calpain-1 expression was observed in 368 (79%) of the 469 available cases (cut-point: 55); whereas, high calpain-2 expression was observed in 379 (81%) of the 469 available cases (cut-point: 10). Low calpain-1 expression was linked with low stage (*χ*^2^ = 15.259, *df* = 3, *P* = 0.002), no residual disease (*χ*^2^ = 15.388, *df* = 2, *P* < 0.001), CCC, endometrioid and mucinous carcinoma (*χ*^2^ = 56.577, *df* = 5, *P* < 0.001) whilst high calpain-1 expression was linked with the presence of residual disease and HGSC (Table 2). Based on tumour stage, patients were grouped according to whether they had an organ-confined tumour (i.e. group 1: stage 1 and group 2: stage 2–4) or according to whether they had distant metastasis (i.e. group 1: stage 1–3 and group 2: stage 4). Calpain-1 showed a significant association with organ-confined ovarian cancers rather than cancers with distant metastases, with low calpain-1 more frequent in organ-confined ovarian cancers (*χ*^2^ = 11.310, *df* = 1, *P* = 0.001). Significant associations between calpain-1 expression and ovarian subtype (*χ*^2^ = 46.754, *df* = 5, *P* < 0.001), stage (*χ*^2^ = 17.449, *df* = 3, *P* = 0.001) and residual disease (*χ*^2^ = 14.723, *df* = 2, *P* = 0.001) were also detected in chemo-naïve samples.

High calpain-4 expression was observed in 326 (70%) out of 469 available cases (cut-point: 95). HGSC was associated with high calpain-4 expression whilst CCC with low calpain-4 expression (*χ*^2^ = 18.181, *df* = 5, *P* = 0.003). This association between calpain-4 and ovarian subtype was also significant in chemo-naïve samples (*χ*^2^ = 15.420, *df* = 5, *P* = 0.009) (Table 2).

### Calpain system expression and clinical outcomes

**Fig. 2:**
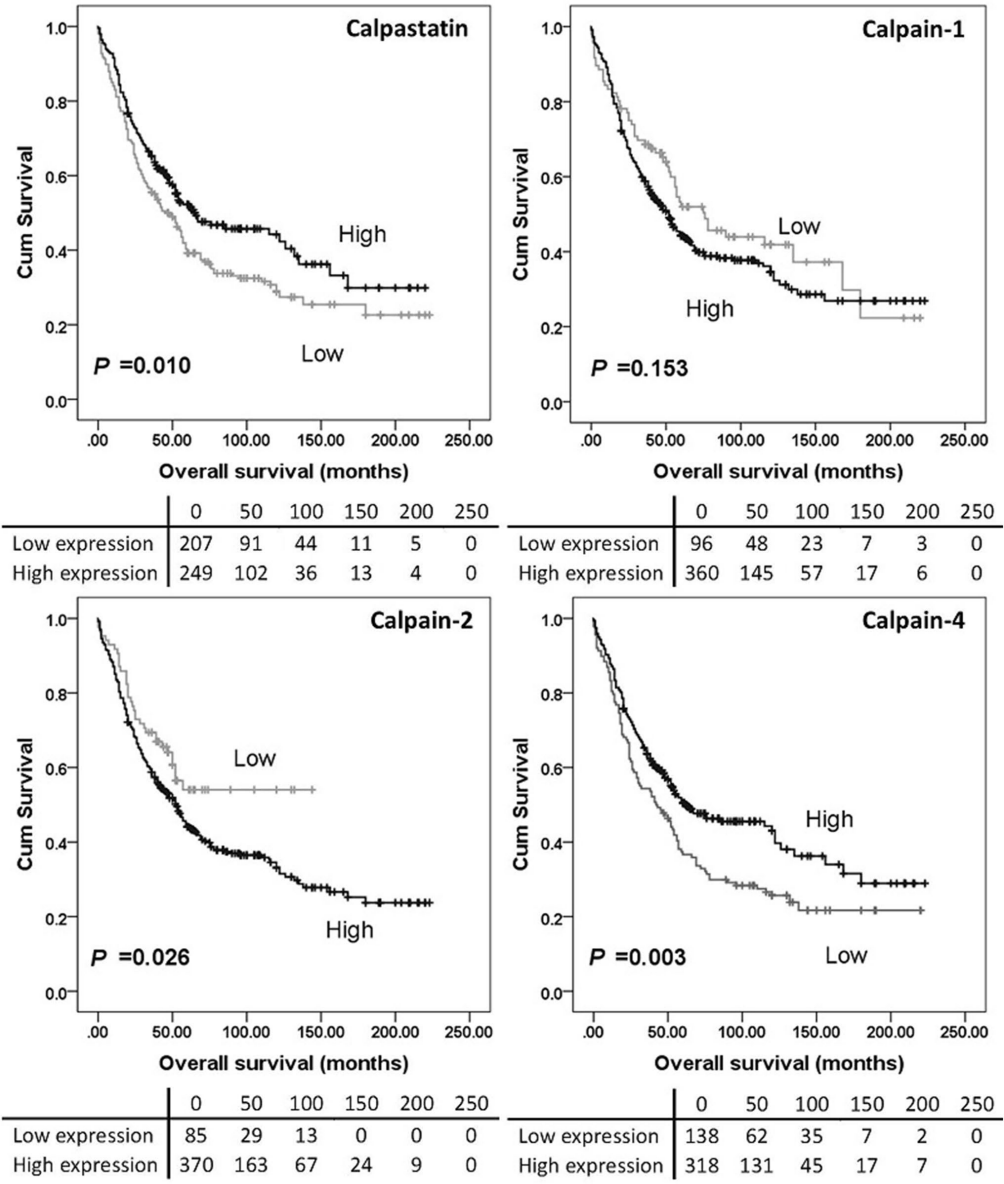
Kaplan–Meier survival curves of calpastatin and calpain-1, -2 and -4 expression, and overall survival. Survival analysis shows significantly better (*P* = 0.010) overall survival for ovarian cancer patients whose tumours express high calpastatin compared to those with tumours expressing low calpastatin. There were no differences in overall survival between patients with tumours expressing high calpain-1 and those with tumours expressing low calpain-1 (*P* = 0.153). Survival analysis showed significantly better (*P* = 0.026) overall survival for ovarian cancer patients whose tumours expressed low calpain-2 compared to those with tumours expressing high calpain-2. Survival analysis also showed significantly better (*P* = 0.003) overall survival for patients with tumours expressing high calpain-4 compared to those expressing low calpain-4. Significance was determined using the log-rank test. The tables shown below the Kaplan–Meier survival curves listed the number of patients at risk at the specific months. High expression—black line, low expression—grey line

**Fig. 3:**
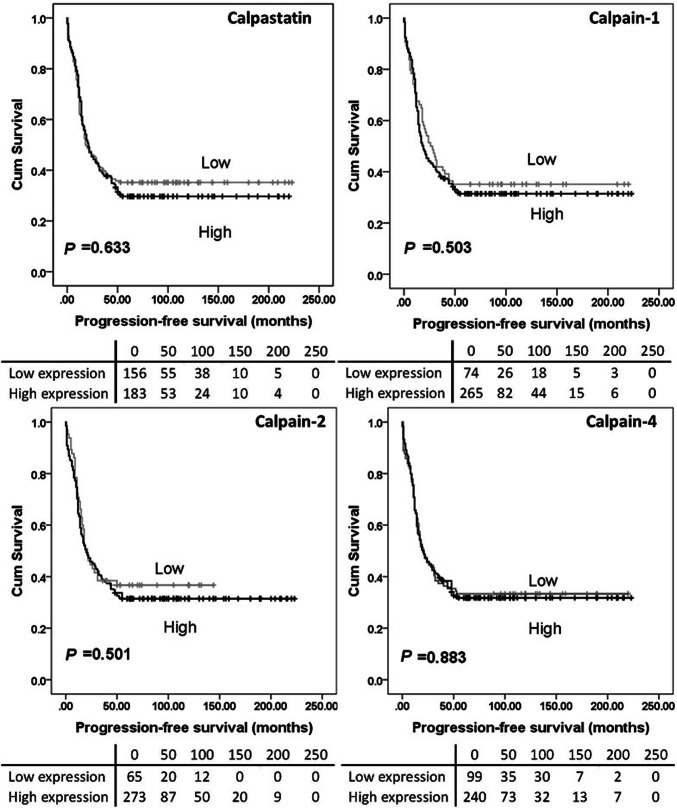
Kaplan–Meier survival curves of calpastatin and calpain-1, -2 and -4 expression, and progression-free survival. Analysis of progression-free survival shows the impact of calpastatin, and calpain-1, -2 and -4 in ovarian cancer patients. Significance was determined using the log-rank test. The tables shown below the Kaplan–Meier survival curves listed the number of patients at risk at the specific months. High expression—black line, low expression—grey line

High calpain-2 expression was significantly associated with adverse overall survival (*P* = 0.026) while high calpain-4 and calpastatin expression were significantly associated with better overall survival (with *P* = 0.003 and *P* = 0.010 respectively) (Fig. 2). No significant association was found between calpain-1 expression and overall survival (*P* = 0.153), and no association was detected between protein expression and progression-free survival (Fig. 3). As all cases (*n* = 154) in the previous study received carboplatin-based adjuvant chemotherapy, a log-rank test was conducted in cases from the current cohort who received carboplatin-based adjuvant chemotherapy (*n* = 352) using the same cut-point calculated from the whole cohort. High calpain-2 was associated with poor overall survival (*P* = 0.030) but not progression-free survival (*P* = 0.381). Calpastatin, calpain-1 and calpain-4 expression were not associated with either overall survival (*P* = 0.129, 0.080 and 0.060, respectively) or progression-free survival (*P* = 0.181, 0.161 and 0.470, respectively) (data not shown).

With *P* values from the log-rank test of less than 0.001, age, grade, FIGO stage, histological subtypes, tumour residue and platinum sensitivity were included in multivariate analysis. Neither calpastatin nor calpain-2/-4 was found as independent markers of overall survival (Table 3).

**Table 3:** Multivariate (Cox proportional hazard regression) analysis

Variables	*P* value	Exp (B)	95% Confidence interval for Exp (B)	
			Lower	Upper
Multivariate (overall survival) (239 patients)				
Age (median 62)	0.225	1.253	0.870	1.805
Grade	0.177	1.445	0.847	2.465
FIGO stage	**0.001**	1.513	1.192	1.919
Histological subtypes	0.833	0.983	0.836	1.155
Platinum sensitivity	< **0.001**	3.142	2.024	4.879
Tumour residue	**0.029**	1.298	1.028	1.639
Calpastatin expression	0.061	0.711	0.498	1.015
Multivariate (overall survival) (238 patients)				
Age (median 62)	0.059	1.404	0.987	1.998
Grade	0.138	1.527	0.873	2.673
FIGO stage	< **0.001**	1.540	1.209	1.962
Histological subtypes	0.959	1.004	0.855	1.180
Platinum sensitivity	< **0.001**	3.272	2.107	5.082
Tumour residue	**0.031**	1.283	1.023	1.610
Calpain-2 expression	0.070	1.524	0.967	2.404
Multivariate (overall survival) (239 patients)				
Age (median 62)	0.164	1.296	0.900	1.867
Grade	0.201	1.427	0.828	2.462
FIGO stage	**0.001**	1.497	1.178	1.904
Histological subtypes	0.801	0.979	0.832	1.152
Platinum sensitivity	< **0.001**	3.088	1.987	4.798
Tumour residue	**0.023**	1.303	1.037	1.639
Calpain-4 expression	0.232	0.793	0.542	1.160

Significant *P* values are indicated by bold type

Exp(B) is used to denote hazard ratio

### Correlations between calpain-1, -2, and -4 and calpastatin expression

**Fig. 4:**
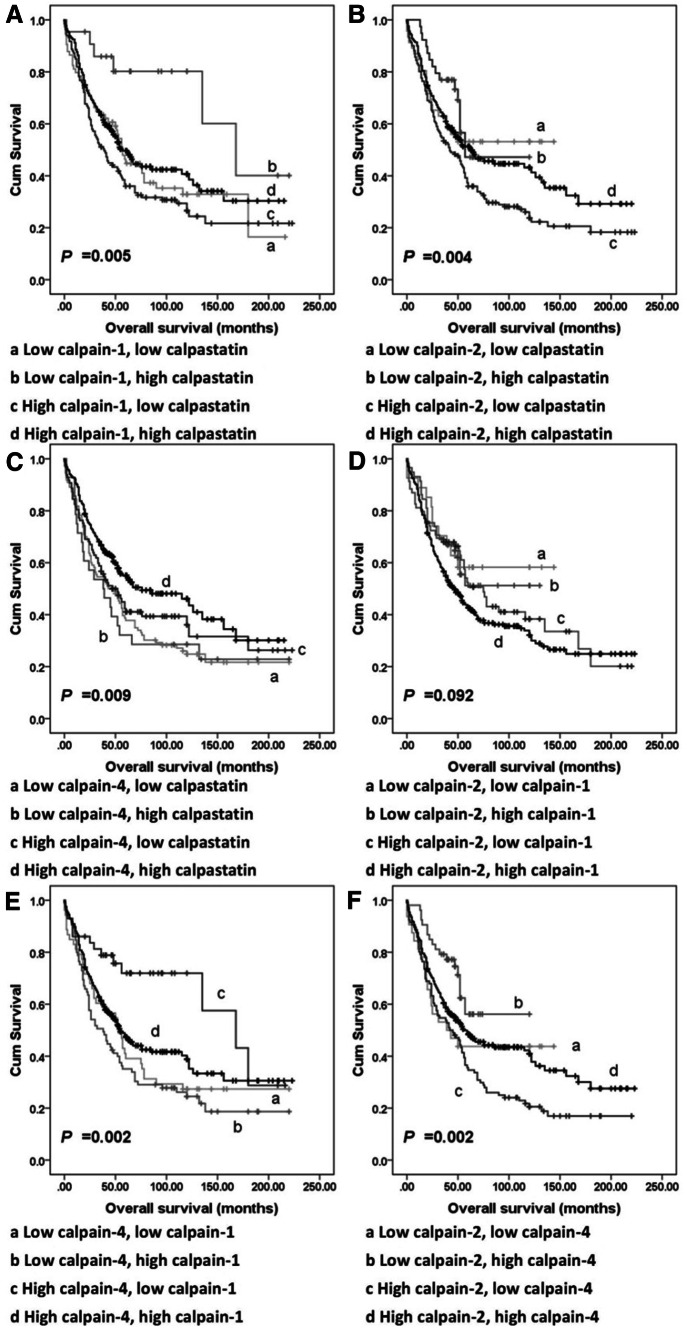
Kaplan–Meier survival curves showing combinatorial calpains and calpastatin expression-related overall survival. **a** survival analysis of combinatorial calpain-1 and calpastatin expression of tumours. **b** survival analysis of combinatorial calpain-2 and calpastatin expression of tumours. **c** survival analysis of combinatorial calpain-4 and calpastatin expression of tumours. **d** Survival analysis of combinatorial calpain-1 and calpain-2 expression of tumours. **e** Survival analysis of combinatorial calpain-1 and calpain-4 expression of tumours. **f** Survival analysis of combinatorial calpain-2 and calpatin-4 expression of tumours. Significance was determined using the log-rank test

Spearman’s rho test was used in assessing the correlation between matched H-scores of conventional calpain subunits and calpastatin. Calpastatin, and calpain-1, -2 and -4 expression were found positively correlated with each other (Table S2). Tumours were then recategorised according to expression of any two of calpain-1, -2, and -4, and calpastatin into four groups each time; for example, using calpain-1 and -2 expression the recategorised groups are: tumour with low expression of both calpain-1 and -2; tumour with high expression of both calpain-1 and -2; tumour with low calpain-1 and high calpain-2 expression; tumour with high calpain-1 and low calpain -2 expression. Significant associations were observed between overall survival and all the combined expression statuses except the combination of calpain-1 and calpain-2 expression (*P* = 0.092; Fig. 4) with none significant from multivariate analysis when conducted as above.

### Calpain expression and overall survival in patient subgroups

Further analysis was conducted to investigate the prognostic significance of conventional calpains and calpastatin in the individual subgroups defined by clinicopathologic variables. The *P* values of Kaplan–Meier survival analysis are summarised in Table S3 with none of the associations statistically significant at a *P* value of 0.001 (set lower due to the multiple testing).

As chemotherapy treatment may induce changes in protein expression in tumours, associations between protein expression and survival outcome were analysed in 448 cases that were chemo-naïve. Calpain-4 (*P* = 0.030) and calpastatin (*P* = 0.022) expression were significantly associated with overall survival; however, the association between calpain-2 expression and overall survival was no longer significant (*P* = 0.532) (data not shown).

### Calpain and calpastatin expression *in vitro* and effects of calpeptin on chemoresponse

**Fig. 5:**
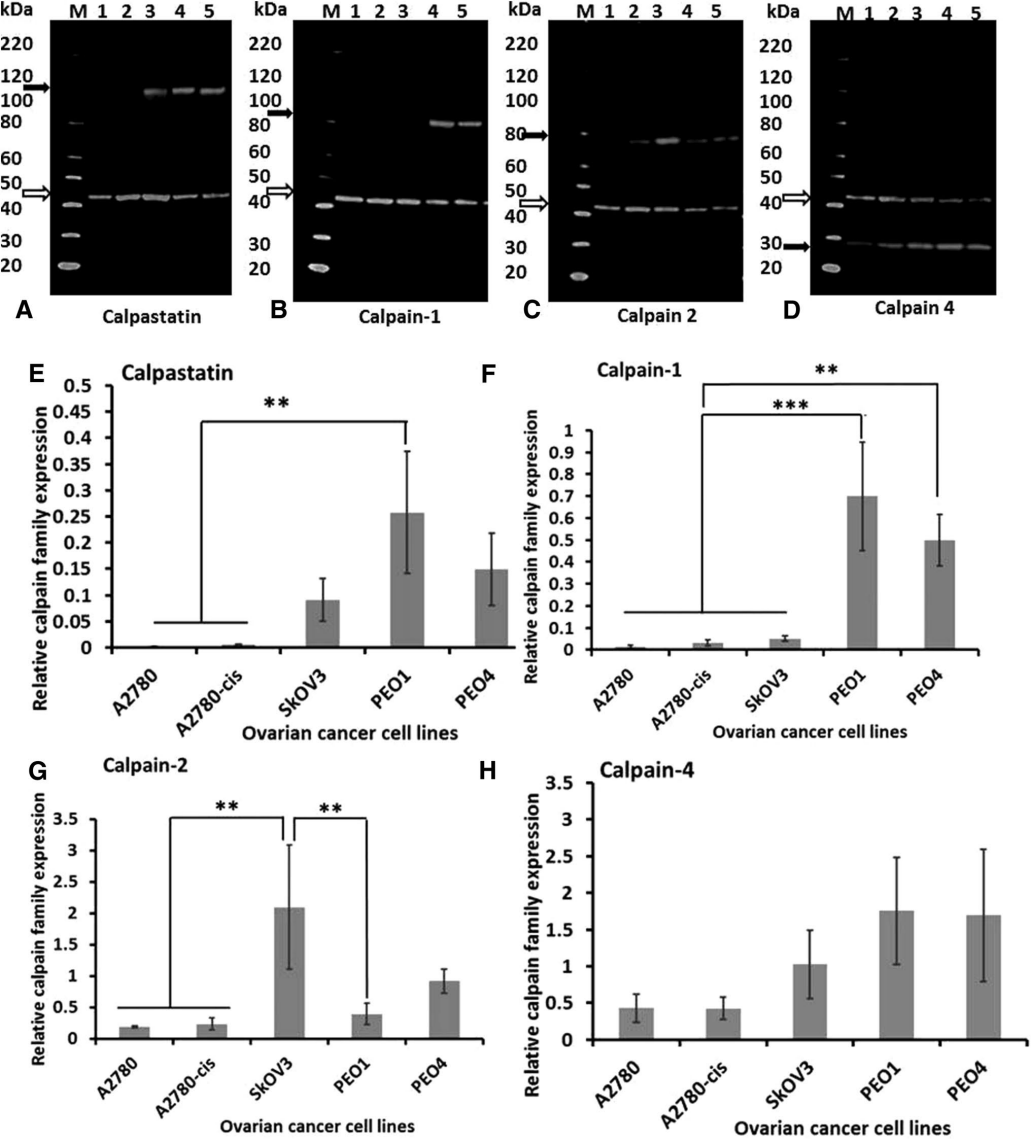
Western blot quantification of protein expression in ovarian cancer cell lines. Representative western blots of three independent experiments are presented. Open arrows indicate β-actin (42 kDa) which was used as the loading control. Black arrows indicate **a** calpastatin (between 100 and 120 kDa), **b** calpain-1 (82 kDa), **c** calpain-2 (80 kDa) and **d** calpain-4 (28-kDa), respectively. Lane M: protein marker, Lane 1: A2780, Lane 2: A2780-cis, Lane 3: SKOV3, Lane 4: PEO1, and Lane 5: PEO4. Graphical representation of protein levels of **e** calpastatin, **f** calpain-1, **g** calpain-2 and **h** calpain-4 in ovarian cancer cell lines relative to β-actin. Data represent the average ± standard deviation of three independent experiments. Statistical significance was determined by one-way ANOVA and is indicated by asterisk. *P < 0.05, **P < 0.01 and ***P < 0.001

To determine if platinum sensitivity could be modulated by altering calpain activity in vitro five ovarian cell lines, with varying sensitivities to platinum-based agents, were used. Protein expression was initially assessed by western blotting (Fig. 5). PEO1 and PEO4 cells expressed higher levels of calpain-1 and -4, and calpastatin than the other three cell lines. Notably, no significant difference was observed between platinum-sensitive cell lines (i.e. A2780 and PEO1) and their resistant counterparts (i.e. A2780-cis and PEO4 respectively). A2780 and A2780-cis cells expressed very low levels of calpain system proteins (Fig. 5) and were more sensitive to calpain inhibition, i.e. at the 48-h time point, IC50 of A2780 and A2780-cis cells were 58 µM and 68 µM whilst IC50 of SKOV3, PEO1 and PEO4 cells were 86 µM, 82 µM and 154 µM. Chemo-sensitisation studies were, therefore, conducted using SKOV3, PEO1 and PEO4 cells.

**Fig. 6:**
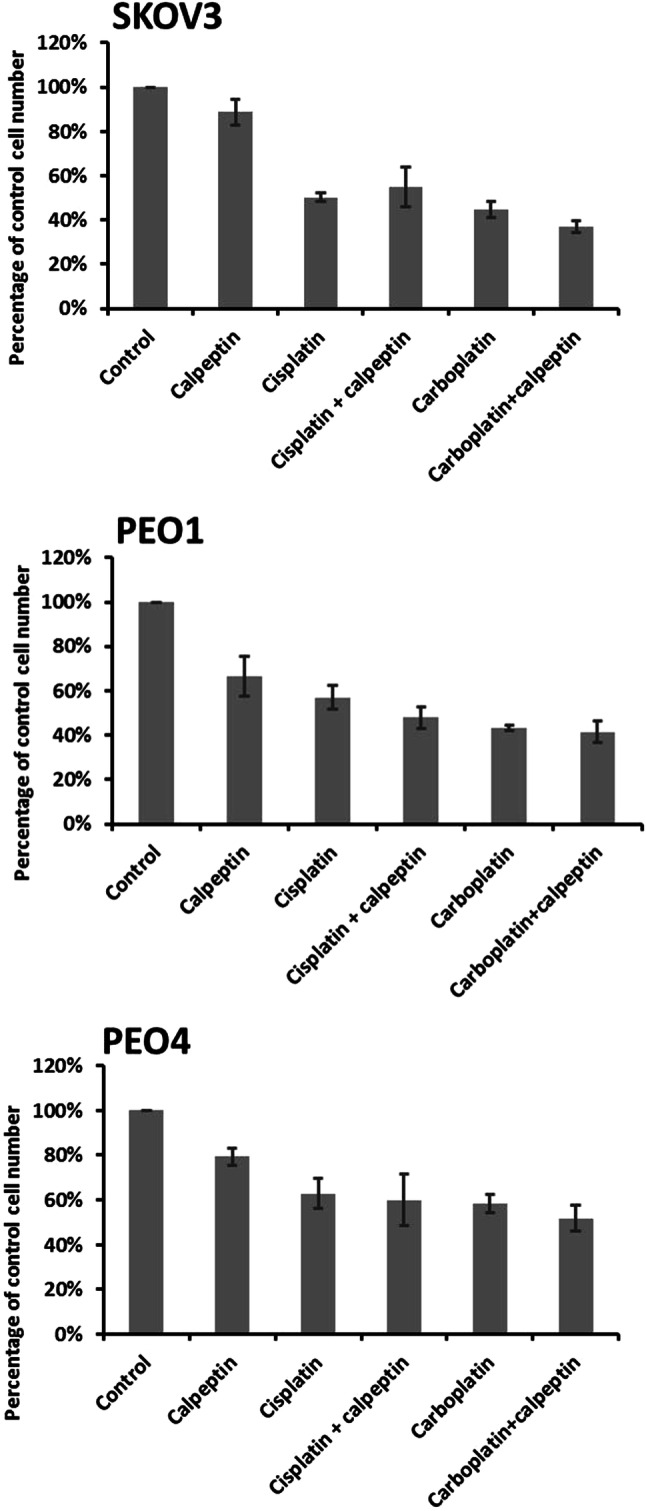
Effect of drug combinations (calpeptin and cisplatin/carboplatin) on cell proliferation. Total cell numbers in drug-treated cultures plotted as a percentage of the vehicle control. Data represent the average ± standard deviation of three independent experiments, with each experiment performed in triplicate

Calpain activity was modulated using the widely used calpain inhibitor calpeptin (Thorpe et al. 2015; Hou et al. 2012). As shown in Fig. 6, use of calpeptin did not alter platinum-based chemotherapy response, as assessed by proliferation, in any of the cell lines used.

### Effect of calpain activity on EMT-related gene expression

RT^2^ Profiler PCR Arrays were used to investigate EMT (epithelial–mesenchymal transition)-related gene expression profiles of PEO1 and PEO4 cells. The comparison of gene expression patterns were first assessed in vehicle control PEO1 and PEO4 cells at 4-h/24-h time point; and then vehicle control group was compared with the respective calpeptin-treated group at each time point in each cell line. When comparing PEO4 with PEO1, BMP7 and COL3A1 genes were shown to be highly differentially expressed (BMP7: 259.84 at 4-h and 351.79 at 24-h–fold, respectively; COL3A1: 75.56 at 4-h and 107.55 at 24-h–fold, respectively), whereas JAG1 and WNT5A genes were expressed in lower levels (JAG1: − 11.73 at 4-h and − 7.15 at 24-h–fold, respectively; WNT5A: -13.74 and − 10.28, respectively). Other differently expressed genes are listed in Table S4. Differences in EMT-related gene expression between vehicle control groups and calpeptin-treated groups were, however, relatively small (fold-change < 10) and may be open to greater variations (threshold cycle > 30). A partial list of the differentially expressed (≥ twofold) EMT-related genes is presented in Table S5.

## Discussion

In the previously published study by our group (*n* = 154), high levels of calpain-2 expression were significantly associated with platinum resistance (*χ*^2^ = 4.658, *df* = 1, *P* = 0.031), adverse progression-free survival (*P* = 0.049) and overall survival (*P* = 0.006) (Storr et al. 2012a), with significance associated with overall survival maintained in multivariate analysis including grade, stage, optimal debulking and platinum sensitivity (hazard ratio = 2.174; 95% confidence interval = 1.144–4.130; *P* = 0.018) (Storr et al. 2012a). The current study, using a larger cohort (*n* = 575), confirms that high calpain-2 expression is significantly associated with worse overall survival (*P* = 0.026). In both studies, calpain-1 was not significantly associated with either progression-free survival (*P* = 0.503) or overall survival (*P* = 0.153). A significant association between calpastatin expression and overall survival was observed in the current study but not the previous one.

µ-Calpain and m-calpain are isozymes, with their large subunits, calpain-1 and calpain-2, sharing the same regulatory subunit calpain-4 (Murachi et al. 1990). From survival analysis, significant associations were observed between overall survival and the combined expression status, except the combination of calpain-1 and calpain-2 expression (*P* = 0.092; Fig. 4). These findings suggest that the role that calpain-1 plays may not be complementary to those of calpain-2.

Although calpain-1 expression was not associated with patient survival, low calpain-1 expression showed significant association with organ-confined status of tumour in the current study. Similar findings were reported by Al-Bahlani et al. (2017) in triple-negative breast cancers; calpain-1 expression was not significantly associated with patient outcome (*n* = 55) but significant association was found between calpain-1 expression and the lymph node status (*P* = 0.02). Significant association has been observed between tumour metastasis (i.e. vessel invasion, presence of lymph node metastasis and/or advanced tumour-node-metastasis stage) and the expression of conventional calpain subunits or calpastatin (Yang et al. 2017; Salehin et al. 2011; Storr et al. 2011b); however, it is context dependent on whether the association was positive or negative. Since stage reflects the extent of ovarian tumour spread in vivo, calpain-1 may be involved in tumour migration and invasion in vitro. Calpain-mediated proteolysis is involved in focal adhesion dynamics and migration in vitro, as reviewed by Franco and Huttenlocher (2005); however, research in this area lacks information on in vivo calpain substrates related to focal adhesions and detachment (Perrin and Huttenlocher 2002). In contrast to our previous data, calpain-2 was not associated with PFS (Fig. 3), whilst calpain-4 and calpastatin was associated with OS (*P* = 0.003 and *P* = 0.010 respectively) (Fig. 2). All cases (*n* = 154) included in the previous study received carboplatin-based adjuvant chemotherapy, thus Kaplan–Meier survival analysis was conducted in 352 cases from the current cohort who received carboplatin-based adjuvant chemotherapy. Again, high calpain-2 was associated with poor OS but no significant association was found between calpain-2 expression and PFS.

The lack of association between calpain-2 and patient response to platinum-based chemotherapy, in the current study, may be due to the variability of polyclonal antibodies between different batches and/or due to the different composition of patients. The percentage of platinum-sensitive patients was different between the previous cohort (67.5%) and the current cohort (82.3%). Patients were then divided into platinum-resistant and platinum-sensitive groups, and log-rank test was conducted in these two groups separately. Conventional calpain subunits and calpastatin did not show significant association with OS in either of these two groups (data not shown). By Pearson’s chi-squared test, neither conventional calpain subunits nor calpastatin was associated with the resistance to platinum-based adjuvant chemotherapy in the current study. Thus, some *in vitro* studies were conducted to help explain the potentially contradictory findings.

Fenouille and colleagues observed different calpain expression levels between the chemo-resistant cells and their parental counterparts (Fenouille et al. 2012); however, in the current in vitro study, chemo-sensitive ovarian cancer cells (i.e. A2780 and PEO1 cells) and their resistant counterparts (i.e. A2780-cis and PEO4 cells, respectively) expressed similar levels of calpain-1, -2, and -4, and calpastatin. Thus, the cisplatin resistance of A2780-cis and PEO4 cells may not be calpain related. In the current study, cells were pre-treated with calpeptin and then exposed to cisplatin or carboplatin. Comparing against cisplatin or carboplatin treatment alone, no altered chemo-sensitisation was detected using cell proliferation as an endpoint. The role of the calpain in regulating chemosensitivity is complex and rather confused. Cisplatin has, in different cancer types, been shown in certain studies to induce apoptosis in a calpain-dependent manner (e.g. melanoma and cervical cancer cells, Del Bello et al. 2013; Shen et al. 2016), with some potentially contradictory findings suggesting that calpain inhibitors can chemo-sensitise certain cancer cells (e.g. colorectal cancer, Fenouille et al. 2012) with others, in hepatoblastoma models (Kim et al. 2007), suggesting that calpains are not among the main regulators of chemotherapy-induced cell death. In ovarian cancer cell lines, calpain activation has been shown to increase following a cisplatin-induced increase of Ca^2+^ levels in OV2008 cells (Al-Bahlani et al. 2011; Woo et al. 2012), whilst in C13* cells (the resistant counterpart of OV2008 cells), Al-Bahlani and colleagues (Al-Bahlani et al. 2011) observed a cisplatin-induced Ca^2+^ level increase and calpain activation, whereas Woo and colleagues (Woo et al. 2012) detected no increased calpain activity.

Recent studies have suggested that the calpain system can play an important role in the process of EMT. In lung cancer cells, for example, transforming growth factor-β1 was showed to induce EMT via calpain-1 (Tan et al. 2017) and in melanoma cells calpain-4 knockdown was able to decrease cell migration and invasion (Wang et al. 2017). Current IHC and in vitro data suggested that the calpain system did not have a major role in chemosensitivity, so the role in regulation of EMT was assessed by expression profiling as alterations to this process may have had a role to play in mediating the decreased OS seen in the patient samples. Calpain inhibition did not cause a large alteration of EMT-related gene expression in ovarian cancer cell lines PEO1 and PEO4 cells. In consideration of the limited fold-change, further verification of the link between calpain activation and EMT-related gene expression needs to be conducted.

One note of caution, to consider, is the limited inhibition achieved by calpeptin, which could be a potential issue of the lack of significant changes in chemosensitivity and EMT-related gene expression of calpeptin-treated cells when compared to the vehicle controls. Calpeptin is a widely used calpain inhibitor. In colorectal cancer cell lines, calpeptin (30 min 100 µM treatment) significantly caused more than 50% inhibition of calpain activity (Thorpe et al. 2015). Autolysis of calpain-1 (i.e. activation) could be significantly suppressed by calpeptin (50 µM for 30 min) in MCF-7 (Hou et al. 2012). Calpeptin (50 µM for 12 h) was also found able to effectively inhibit calpain-induced IκBα depolymerisation in MCF7 cells (Kim et al. 2010). Immunoblotting results indicated that calpeptin (40 µM for 24 h) effectively inhibited calpain activity, and protected filamin A and androgen receptor from cleavage by calpain in human prostate cancer cell lines PC-3 (Liu et al. 2014). Such data suggest that 40–100 µM calpeptin treatment for at least 30 min could effectively inhibit calpain activity and calpain-induced protein cleavage. In the current study, a commonly used dose of 50 µM calpeptin induced a 30–40% inhibition with wide variation and increasing the dose did not increase inhibition but did start to induce cytotoxic effects (data not shown).

As PEO1 was derived from ascitic fluid of a patient with ovarian adenocarcinoma whilst PEO4 was derived from the ascites of the same patient at the time of relapse the COL3A, and BMP7 differential expression, in the current study, may be of interest in terms of tumour progression. Very limited high-throughput screening studies have been conducted on EMT-related proteins in relation to ovarian cancer. A similar tendency, as seen in the current study, with COL3A1 protein was seen in a previous study in that expression was higher in a highly proliferative ovarian carcinoma cell line than in a low malignant potential one (Gagné et al. 2007). COL3A1 gene has also been found differentially expressed between primary ovarian carcinomas and metastases (Li et al. 2017). Le Page and colleagues showed that BMP2 was differentially transcribed between cells derived from ascites and solid tumours (Le Page et al. 2006) whilst Hibbs and colleagues suggested BMP7 was more highly expressed in ovarian carcinomas than normal ovaries, but was unable to distinguish between ovarian tumour tissues and metastasis (Hibbs et al. 2004). Various studies have suggested both tumour-promoting and suppressive roles of BMPs in tumour progression in a wide range of human tumours depending on the status of tumours and their microenvironment (Bach et al. 2017). Thus, the exact role of BMPs in ovarian cancer, and resistance to platinum-based chemotherapeutic drugs, requires further investigation.

In conclusion, both the previous and the current ovarian cancer patient-based studies indicate that calpain-2 expression is adversely associated with overall survival, with calpain-4 and calpastatin expression also negatively associated with overall survival in the current study. In ovarian cancers, the calpain system has been confirmed to play an important role and influence patient outcome; however, the precise mechanisms whereby it exerts effects remain to be elucidated.

## Electronic supplementary material

Below is the link to the electronic supplementary material.

Supplementary material 1 (PDF 336 KB)

## Abbreviations

EMT: Epithelial–mesenchymal transition
CCC: Clear-cell carcinoma
dH_2_O: Distilled water
DMSO: Dimethyl sulfoxide
HGSC: High-grade serous carcinoma
ICC: Intraclass correlation coefficient
LGSC: Low-grade serous carcinoma
RPMI: Roswell Park Memorial Institute
rs: Spearman rho
SBOT: Serous borderline ovarian tumour

## References

[CR1] Al-Bahlani S, Fraser M, Wong AY et al (2011) P73 regulates cisplatin-induced apoptosis in ovarian cancer cells via a calcium/calpain-dependent mechanism. Oncogene 30(41):4219–4230 21516125 10.1038/onc.2011.134 PMC3194400

[CR2] Al-Bahlani SM, Al-Rashdi RM2, Kumar S et al (2017). Calpain-1 expression in triple-negative breast cancer: a potential prognostic factor independent of the proliferative/apoptotic index. Biomed Res Int. 2017:9290425 10.1155/2017/9290425 PMC5425834 28536704

[CR3] Bach DH, Park HJ, Lee SK (2017) The dual role of bone morphogenetic proteins in cancer. Mol Ther Oncolytics 8:1–13. 10.1016/j.omto.2017.10.002 29234727 10.1016/j.omto.2017.10.002 PMC5723373

[CR4] Camp RL, Dolled-Filhart M, Rimm DL (2004) X-tile: a new bio-informatics tool for biomarker assessment and outcome-based cut-point optimization. Clin Cancer Res 10(21):7252–7259 15534099 10.1158/1078-0432.CCR-04-0713

[CR5] Del Bello B, Toscano M, Moretti D, Maellaro E (2013) Cisplatin-induced apoptosis inhibits autophagy, which acts as a pro-survival mechanism in human melanoma cells. PLoS One 8(2):e57236 23437349 10.1371/journal.pone.0057236 PMC3577730

[CR6] Divaris K, Vann WF Jr, Baker AD, Lee JY (2012) Examining the accuracy of caregivers’ assessments of young children’s oral health status. J Am Dent Assoc 143(11):1237–1247 23115154 10.14219/jada.archive.2012.0071 PMC3697431

[CR7] Fenouille N, Grosso S, Yunchao S et al (2012) Calpain 2-dependent IκBα degradation mediates CPT-11 secondary resistance in colorectal cancer xenografts. J Pathol 227(1):118–129 22069124 10.1002/path.3034

[CR8] Franco SJ, Huttenlocher A (2005) Regulating cell migration: Calpains make the cut. J Cell Sci 118(Pt 17):3829–3838 16129881 10.1242/jcs.02562

[CR9] Gagné JP, Ethier C, Gagné P et al (2007) Comparative proteome analysis of human epithelial ovarian cancer. Proteome Sci 5:16 17892554 10.1186/1477-5956-5-16 PMC2072939

[CR10] Hibbs K, Skubitz KM, Pambuccian SE et al (2004) Differential gene expression in ovarian carcinoma: identification of potential biomarkers. Am J Pathol 165(2):397–414 15277215 10.1016/S0002-9440(10)63306-8 PMC1618570

[CR11] Hou J, Wang X, Li Y et al (2012) 17beta-estradiol induces both up-regulation and processing of cyclin E in a calpain-dependent manner in MCF-7 breast cancer cells. FEBS Lett 586(6):892–896 22449977 10.1016/j.febslet.2012.02.018

[CR12] Kim MJ, Oh SJ, Park SH et al (2007) Hypoxia-induced cell death of HepG2 cells involves a necrotic cell death mediated by calpain. Apoptosis 12(4):707–718 17195093 10.1007/s10495-006-0002-3

[CR13] Kim DS, Han BG, Park KS et al (2010) I-kappaBalpha depletion by transglutaminase 2 and mu-calpain occurs in parallel with the ubiquitin-proteasome pathway. Biochem Biophys Res Commun 399(2):300–306 20659425 10.1016/j.bbrc.2010.07.078

[CR14] Le Page C, Ouellet V, Madore J et al (2006) Gene expression profiling of primary cultures of ovarian epithelial cells identifies novel molecular classifiers of ovarian cancer. Br J Cancer 94(3):436–445 16421595 10.1038/sj.bjc.6602933 PMC2361148

[CR15] Li S, Li H, Xu Y, Lv X (2017) Identification of candidate biomarkers for epithelial ovarian cancer metastasis using microarray data. Oncol Lett 14(4):3967–3974 28943904 10.3892/ol.2017.6707 PMC5604128

[CR16] Liu T, Mendes DE, Berkman CE (2014) Prolonged androgen deprivation leads to overexpression of calpain 2: implications for prostate cancer progression. Int J Oncol 44(2):467–472 24297527 10.3892/ijo.2013.2196 PMC3898865

[CR17] McShane LM, Altman DG, Sauerbrei W et al (2005) REporting recommendations for tumour MARKer prognostic studies (REMARK). Br J Cancer 93:387–391 16106245 10.1038/sj.bjc.6602678 PMC2361579

[CR18] Młynarczuk-Biały I, Roeckmann H, Kuckelkorn U et al (2006) Combined effect of proteasome and calpain inhibition on cisplatin-resistant human melanoma cells. Cancer Res 66(15):7598–7605 16885359 10.1158/0008-5472.CAN-05-2614

[CR20] Murachi T, Adachi Y, Hatanaka M et al (1990) Gene expression for calpain isozymes in human hematopoietic system cells. Prog Clin Biol Res 344:477–494 2203051

[CR21] Perrin BJ, Huttenlocher A (2002) Calpain. Int J Biochem Cell Biol 34(7):722–725 11950589 10.1016/s1357-2725(02)00009-2

[CR22] Salehin D, Fromberg I, Haugk C et al (2011) Immunohistochemical analysis for expression of calpain 1, calpain 2 and calpastatin in ovarian cancer. Eur J Gynaecol Oncol 32(6):628–635 22335024

[CR23] Shen L, Wen N, Xia M et al (2016) Calcium efflux from the endoplasmic reticulum regulates cisplatin-induced apoptosis in human cervical cancer HeLa cells. Oncol Lett 11(4):2411–2419 27073489 10.3892/ol.2016.4278 PMC4812401

[CR24] Storr SJ, Carragher NO, Frame MC et al (2011a) The calpain system and cancer. Nat Rev Cancer 11(5):364–374 21508973 10.1038/nrc3050

[CR25] Storr SJ et al (2011b) Calpastatin is associated with lymphovascular invasion in breast cancer. Breast 20(5):413–418 21531560 10.1016/j.breast.2011.04.002

[CR26] Storr SJ, Woolston CM, Barros FF et al (2011c) Calpain-1 expression is associated with relapse-free survival in breast cancer patients treated with trastuzumab following adjuvant chemotherapy. Int J Cancer 129(7):1773–1780 21140455 10.1002/ijc.25832

[CR27] Storr SJ, Safuan S, Woolston CM et al (2012a) Calpain-2 expression is associated with response to platinum based chemotherapy, progression-free and overall survival in ovarian cancer. J Cell Mol Med 16(10):2422–2428 22435971 10.1111/j.1582-4934.2012.01559.x PMC3472029

[CR28] Storr SJ, Zaitoun AM, Arora A et al (2012b) Calpain system protein expression in carcinomas of the pancreas, bile duct and ampulla. BMC Cancer 12:511 23140395 10.1186/1471-2407-12-511 PMC3542103

[CR29] Tan WJ, Tan QY, Wang T et al (2017) Calpain 1 regulates TGF-β1-induced epithelial-mesenchymal transition in human lung epithelial cells via PI3K/Akt signaling pathway. Am J Transl Res 9(3):1402–1409. eCollection 2017 28386365 PMC5376030

[CR30] Thorpe H, Akhlaq M, Jackson D et al (2015) Multiple pathways regulate Cten in colorectal cancer without a Tensin switch. Int J Exp Pathol 96(6):362–369 26852686 10.1111/iep.12154 PMC4744826

[CR31] Wang E, Wang D, Li B et al (2017) Capn4 promotes epithelial-mesenchymal transition in human melanoma cells through activation of the Wnt/β-catenin pathway. Oncol Rep 37(1):379–387 27878263 10.3892/or.2016.5247

[CR32] Woo MG, Xue K, Liu J et al (2012) Calpain-mediated processing of p53-associated parkin-like cytoplasmic protein (PARC) affects chemosensitivity of human ovarian cancer cells by promoting p53 subcellular trafficking. J Biol Chem 287(6):3963–3975 22117079 10.1074/jbc.M111.314765 PMC3281727

[CR33] Yang X, Sun J, Xia D et al (2017) Capn4 enhances osteopontin expression through activation of the Wnt/β-catenin pathway to promote epithelial ovarian carcinoma metastasis. Cell Physiol Biochem 42(1):185–197 28535511 10.1159/000477310

[CR34] Zhang Y, Xu W, Ni P et al (2016) MiR-99a and MiR-491 regulate cisplatin resistance in human gastric cancer cells by targeting CAPNS1. Int J Biol Sci 12(12):1437–1447. eCollection 2016 27994509 10.7150/ijbs.16529 PMC5166486

[CR35] Zhang Y, Liu NM, Wang Y et al (2017) Endothelial cell calpain as a critical modulator of angiogenesis. Biochim Biophys Acta 1863(6):1326–1335 10.1016/j.bbadis.2017.03.021 PMC6511073 28366876

