# Supplementary material for: Calpain system protein expression and activity in ovarian cancer
Source: J Cancer Res Clin Oncol. 2018 Nov 17;145(2):345–61. doi: 10.1007/s00432-018-2794-2 (PMC6373250; doi:10.1007/s00432-018-2794-2)
Supplement: Supplementary file 1 — Supplementary material 1 (PDF 336 KB) [file 432_2018_2794_MOESM1_ESM.pdf]

Calpain system protein expression and activity in ovarian cancer

Journal of Cancer Research and Clinical Oncology

Siwei Zhang[1], Suha Deen[2], Sarah J Storr[1], Panagiota S Chondrou[1,3], Holly Nicholls[1], Anqi Yao[1], Ployphailin Rungsakaolert[1], Stewart G Martin[1,4]

1 Academic Clinical Oncology, Nottingham Breast Cancer Research Centre, School of Medicine, University of Nottingham, Nottingham University Hospitals NHS Trust, City Hospital Campus, Nottingham, NG5 1PB, UK.
2 Department of Histopathology, Queen's Medical Centre, Nottingham University Hospitals NHS Trust, Nottingham, NG7 2UH, UK
3 Current address: Lungs for Living Research Centre, UCL Respiratory, University College London, 5 University Street London, WC1E 6JF United Kingdom.
4 Corresponding author: Academic Clinical Oncology, Nottingham Breast Cancer Research Centre, School of Medicine, University of Nottingham, Nottingham University Hospitals NHS Trust, City Hospital Campus, Nottingham, NG5 1PB, UK
Telephone: +44(0)115 823 1846
Email: stewart.martin@nottingham.ac.uk.

Supplements

Table S1 Treatment received by the ovarian cancer patients in present study

Treatment	Number of Patients	Frequency (%)*
Carboplatin	148	25.7
Carboplatin and paclitaxel (taxol ®)	160	27.8
Carboplatin and docetaxel (Taxotere ®)	2	0.3
Carboplatin, docetaxel and irinotecan (Campto ®)	1	0.2
Carboplatin, paclitaxel and topotecan (Hycamtin ®)	3	0.5
Carboplatin, paclitaxel and bevacizumab	1	0.2
Carboplatin and cyclophosphamide	5	0.9
Carboplatin, cyclophosphamide and doxorubicin (adriamycin)	21	3.7
Carboplatin and topotecan	9	1.6
Carboplatin and radiotherapy (HGSC)	1	0.2
Carboplatin, paclitaxel and radiotherapy (HGSC)	1	0.2
CAP: cyclophosphamide, doxorubicin and cisplatin	5	0.9
Chlorambucil (Leukeran ®)	2	0.3
Topotecan (Hycamtin ®)	2	0.3
Gemcitabine (Gemzar ®)	1	0.2
No chemotherapy	74	12.9
Radiotherapy (HGSC and endometrioid carcinoma)	2	0.3
Radiotherapy and progesterone (endometrioid carcinoma)	1	0.2
Dexamethasone and radiotherapy for lung metastasis (endometrioid carcinoma)	1	0.2
BIBF 1120 (Nintedanib)	2	0.3

* Percentage of the total cohort (n=575).

Table S2 Spearman's rank correlation coefficient between calpain-1, -2, -4 and calpastatin expression

		Calpain-1	Calpain-2	Calpain-4
**Calpastatin**	**Correlation Coefficient**	**0.480****	**0.205****	**0.514****
	**Sig. (2-tailed)**	2.4147E-28	0.000008	6.783E-33
**Calpain-1**	**Correlation Coefficient**	\	**.300****	**0.481****
	**Sig. (2-tailed)**	\	3.3557E-11	1.5002E-28
**Calpain-2**	**Correlation Coefficient**	\	\	**0.296****
	**Sig. (2-tailed)**	\	\	6.4717E-11

** Correlation is significant at the 0.01 level (2-tailed).

* Correlation is significant at the 0.05 level (2-tailed).

Significant P-values are indicated by bold type.

Table S3 Kaplan–Meier survival analysis in different subgroups of ovarian cancer

	***P* value (confidence interval: 99%)**			
	Calpastatin	Calpain-1	Calpain-2	Calpain-4
**Histological subtypes**				
**HGSC (n=265)**	0.002	0.658	0.011	0.064
**Mucinous carcinoma (n=45)**	0.497	0.448	0.556	0.014
**Endometrioid carcinoma (n=53)**	0.415	0.617	0.713	0.899
**CCC (n=41)**	0.564	0.308	0.826	0.461
**LGSC (n=24)**	0.930	0.866	0.257	0.987
**Stage**				
**Stage 1 (confined tumour) (n=163)**	0.019	0.997	0.634	0.011
**Stage 2, 3 & 4 (tumour spread) (n=286)**	0.008	0.766	0.025	0.044
**Stage 1, 2 & 3 (without distant metastasis) (n=416)**	0.019	0.195	0.078	0.004
**Stage 4 (with distant metastasis) (n=33)**	0.976	0.254	0.024	0.963
**Sensitivity to platinum-based chemotherapy**				
**Sensitive (n=213)**	0.050	0.049	0.435	0.036
**Resistant (n=39)**	0.798	0.815	0.076	0.433
**Residual disease**				
**No residual tumour (n=250)**	0.085	0.988	0.244	0.026
**residual tumour (n=153)**	0.002	0.361	0.594	0.012

Table S4 Profiler PCR array results (PEO4 cells vs. PEO1 cells).

Refseq	Gene	Description	Fold-change			
			4h		24h	
PPH00527A	BMP7	Bone morphogenetic protein 7	259.84	A	351.79	A
PPH00439F	COL3A1	Collagen, type III, alpha 1 chain precursor	75.56	A	107.55	A
PPH00152E	MMP9	Matrix metallopeptidase 9	4.38	A	2.88	B
PPH20529A	NUDT13	Nudix (nucleoside diphosphate linked moiety X)-type motif 13	4.17	A	3.53	B
PPH01944B	NODAL	Nodal homolog (mouse)	11.16	B	16.07	B
PPH09021B	ZEB2	Zinc finger E-box binding homeobox 2	2.11	B	11.02	B
PPH00781A	COL5A2	Collagen, type V, alpha 2	4.47	B	7.66	B
PPH02389A	KRT14	Keratin 14	5.22	B	5.40	B
PPH00215F	SERPINE1	Serpin peptidase inhibitor, clade E, member 1	4.68	B	\	\
PPH00235F	MMP3	Matrix metallopeptidase 3	4.24	B	\	\
PPH02459B	SNAI1	Snail homolog 1 (Drosophila)	3.22	B	\	\
PPH02399C	WNT11	Wingless-type MMTV integration site family, member 11	3.15	B	2.57	B
PPH00531F	TGFB3	Transforming growth factor, beta 3	2.72	B	\	\
PPH00526C	NOTCH1	Notch 1	2.48	B	\	\
PPH11409A	TMEM132A	Transmembrane protein 132A	6.29	A	-2.27	A
PPH02410A	WNT5A	Wingless-type MMTV integration site family, member 5A	-13.74	A	-10.28	A
PPH06022B	JAG1	Jagged 1	-11.73	A	-7.15	A
PPH00524B	TGFB2	Transforming growth factor, beta 2	-2.05	\	-9.22	A
PPH02475A	SNAI2	Snail homolog 2 (Drosophila)	-2.72	\	-6.98	A
PPH02447C	WNT5B	Wingless-type MMTV integration site family, member 5B	-5.32	\	-5.47	A
PPH00176C	ITGA5	Integrin, α 5 (fibronectin receptor)	-3.90	A	-2.25	A
PPH00582E	SPP1	Secreted phosphoprotein 1	\	\	-3.23	A
PPH00515B	BMP1	Bone morphogenetic protein 1	-2.86	A	-2.41	A
PPH00555G	IL1RN	Interleukin 1 receptor antagonist	-2.70	A	-2.44	A
PPH00636F	CDH2	N-cadherin	-2.21	A	-17.91	B
PPH00151B	MMP2	Matrix metallopeptidase 2	-13.11	B	-8.84	B
PPH01971A	FOXC2	Forkhead box C2	-5.70	B	\	\
PPH01922A	ZEB1	Zinc finger E-box binding homeobox 1	\	\	-3.85	B
PPH15155C	SNAI3	Snail homolog 3 (Drosophila)	-2.02	B	\	\

A: This gene's expression is relatively low (threshold cycle > 30) in one sample and reasonably detected (threshold cycle < 30) in the other sample suggesting that the actual fold-change value is at least as large as the calculated and reported fold-change result.

B: This gene's relative expression level is low (threshold cycle > 30), in both control and test samples.

Table S5 Profiler PCR array results for PEO1 and PEO4 cells (calpeptin-treated group vs. vehicle control group at two time points).

Gene	Description	Fold-change	
**Up-regulated gene expression in PEO1**		**4h**	**24h**
**ZEB2**	Zinc finger E-box binding homeobox 2	3.21 (B)	4.32 (B)
**BMP2**	Bone morphogenetic protein 2	\	2.71 (B)
**BMP7**	Bone morphogenetic protein 7	2.04 (B)	\
**Up-regulated genes in PEO4**		**4h**	**24h**
**CDH2**	Cadherin 2, type 1, N-cadherin (neuronal)	\	8.76 (B)
**KRT14**	Keratin 14	2.72(B)	2.18 (B)
**MMP2**	Matrix metallopeptidase 2 (gelatinase A, 72kDa gelatinase, 72kDa type IV collagenase)	2.40(B)	\
**SNAI3**	Snail homolog 3 (Drosophila)	2.39(B)	\
**ZEB2**	Zinc finger E-box binding homeobox 2	2.37(B)	\
**Down-regulated gene expression in PEO1**		**4h**	**24h**
**MMP9**	Matrix metallopeptidase 9 (gelatinase B, 92kDa gelatinase, 92kDa type IV collagenase)	-3.06 (B)	\
**NOTCH1**	Notch 1	-2.20 (B)	\
**Down-regulated gene expression in PEO4**		**4h**	**24h**
**NODAL**	Nodal homolog (mouse)	-2.99 (B)	-9.83 (B)
**WNT11**	Wingless-type MMTV integration site family, member 11	-2.03 (B)	-2.06 (B)
**ZEB1**	Zinc finger E-box binding homeobox 1	-2.40 (B)	
**ZEB2**	Zinc finger E-box binding homeobox 2		-4.71 (B)
